# Teaching Cell and Gene Therapy in Genetic Counseling Programs: Survey Findings and Suggested Practical Resources for Integration

**DOI:** 10.1002/jgc4.70295

**Published:** 2026-09-22

**Authors:** Mariel Sebby, Lily McConachie, Leah Wetherill, Paula Delk, Emily L. Hopewell

**Affiliations:** ^1^ Department of Medical and Molecular Genetics Indiana University School of Medicine Indianapolis Indiana USA; ^2^ Department of Medical and Molecular Genetics Indiana University Health Indianapolis Indiana USA; ^3^ Brown Center for Immunotherapy Indiana University School of Medicine Indianapolis Indiana USA; ^4^ Indiana University Melvin and Bren Simon Comprehensive Cancer Center, Indiana University School of Medicine Indianapolis Indiana USA

**Keywords:** barriers, cell therapy, education, facilitators, gene therapy, genetic counseling, resources

## Abstract

As cell and gene therapies (CGTs) advance through clinical trials into clinical practice, it becomes increasingly important for genetic counselors (GCs) to understand these therapeutics to discuss them with patients. Despite the apparent need for GCs to obtain CGT‐related education, little is known about the training GCs receive on CGT topics in their graduate training. To address this, we performed a cross‐sectional quantitative survey of North American genetic counseling training programs. The survey inquired about the extent of training on CGT‐related topics as well as barriers and facilitators that influence such training. Leadership from 45 of 65 (69.2%) genetic counseling programs completed the survey. Most currently provide some form of CGT training, although for more than half of the programs, it is relatively new content added to their curriculum within the past 5 years. Most programs provide training on all subtopics explored; however, the teaching methodology used and the number of exposures in each program varied. The most frequent barrier to adding training was lack of time in students' schedules (28.9%) and the most frequent facilitator was access to professionals in the CGT field (53.3%). Despite the consensus that CGT education is important, this study found that many factors influence the robustness of a program's training on these topics, including a lack of consistency in educational content and training modalities among graduate programs. These results and those of previous studies suggest that a centralized resource available to GC students and program leadership could increase access to information about CGT and improve education in this area.

## Abstract (highlights)

**What Is Known About This Topic:**

As more cell and gene therapies (CGTs) for genetic conditions are approved each year, it is increasingly important for genetic counselors to have a strong knowledge base in CGT to effectively discuss these options with patients. Previous studies suggest that genetic counselors may not be receiving adequate education on CGTs or do not remember their training.

**What This Paper Adds to the Topic:**

This study surveyed genetic counseling graduate programs to explore CGT education during graduate training and to identify the barriers and facilitators that influence whether programs offer this education. Our findings suggest that while most genetic counseling programs provide some training on this topic, the depth of training may be limited. These results, combined with recent publications, suggest that a practice resource accessible to all genetic counselors and training programs may help increase accessibility and provide an appropriate foundation of knowledge regarding CGT.

## Plain Language Summary

This study found that most genetic counseling programs teach about cell and gene therapy (CGT), but the amount and type of training are not the same in every program. Our study also found that lack of time in a student's schedule makes it hard to add training on CGT, while access to CGT professionals makes it easier to add this training for students.

## 1 Introduction

Cell and gene therapy (CGT) involves treatments that either replace or modify cells or genes to mitigate disease. The US Food and Drug Administration (FDA) has approved a number of CGTs for a variety of cancers, including both liquid and solid tumors, and for monogenic diseases such as sickle cell disease and hemophilia (*Gene Therapy Program | FDA‐Approved Gene Therapies | Boston Children's Hospital* 2026). Additionally, ongoing clinical trials continue to investigate new therapies that could become additional viable treatment options for patients with genetic conditions such as Fabry disease (NCT04519749, NCT04999059) and Duchenne Muscular Dystrophy (NCT03333590, NCT05693142) (Cring and Sheffield 2022). In November 2025, the Alliance for Regenerative Medicine reported 2125 CGT clinical trials in progress in North America, Asia Pacific, and Europe (*Q3 2025 Cell and Gene Therapy Sector Snapshot* 2025).

Genetic counselors (GCs) play a key role in conveying complex information and fostering informed decisions for patients regarding clinical trials and research opportunities (Bloom et al. 2025). The rapid expansion of CGT as a viable treatment option, coupled with the fact that many businesses are selling non‐FDA approved stem cell therapies that lack safety and efficacy data (Turner 2021), makes it imperative to train GCs to identify safe, properly regulated CGT clinical trials and approved CGTs and to accurately discuss these treatment options with their patients. The scope of practice for GCs, outlined by the National Society of Genetic Counselors, notes a GC's role in discussing management for genetic conditions (*National Society of Genetic Counselors* 2026). While not specifically detailed, counseling about CGTs and informing patients about available clinical trials and therapies falls within this scope of practice. Regardless of subspecialty, GCs can and should be discussing approved therapies with patients when applicable as well as facilitating the decision‐making process with those considering these therapies (Beretich and Beretich 2023; Bloom et al. 2025; Vockley et al. 2023).

More broadly, in November 2021, a global advisory board meeting on the topic of gene therapy was held, consisting of attendees selected from major international medical genetics societies. The advisory board proposed that GCs should be trained to educate and counsel patients on the basics of gene therapy with the call to action to take place in the next 1–3 years (Vockley et al. 2023). Increasing GCs' comfort and expertise in counseling about CGTs can enable GCs to work at the top of their scope, providing comprehensive care that reflects the growing integration of these advancements into clinical practice.

Despite the clear need for CGT knowledge among GCs, little is currently known about the implementation of training on CGT‐related topics within genetic counseling graduate programs. A previous study found that many GCs felt their knowledge on CGT was not sufficient to address patient questions (Geiselman et al. 2024), implying a potential gap in the training of GCs. Only 28% of respondents agreed that the coverage of CGT in their training programs was adequate (Geiselman et al. 2024). This may indicate that of the more than 8000 American Board of Genetic Counselors (ABGC) certified GCs in North America, over 5000 may feel that they do not have adequate training to address patient questions around CGT (*Genetic Counselor Workforce* 2026). Another recent study reported that although 38% of respondents remembered their genetic counseling training program covering topics related to gene therapy, 83% responded that they wanted more training (Walsh et al. 2024). Together, these studies suggest that understanding the scope of CGT education included in training programs is imperative.

The primary objective of this study was to describe the extent of training on CGT‐related topics in genetic counseling training programs in the United States and Canada. Our secondary goal was to identify barriers that limit and factors that facilitate CGT training. These results may identify actionable factors that can be addressed to improve CGT education within program curricula. As CGT trials and approved CGTs become more prevalent, it is crucial that training programs educate future GCs on understanding the utility of CGTs for patients and how to identify safe CGT trials and approved CGTs.

## 2 Methods

### 2.1 Participants

This study was deemed exempt by the Indiana University Institutional Review Board (Protocol #27284). Participants were recruited through the Genetic Counselor Educators Association (GCEA) listserv, which consists of program leaders from all genetic counseling graduate programs in North America. Eligibility criteria required participants to hold a leadership role in a genetic counseling graduate school training program that had successfully graduated at least one cohort by June 2025, when the initial survey was distributed. According to the Accreditation Council for Genetic Counseling (ACGC), at the time the study was conducted there were 65 accredited genetic counseling programs across the United States and Canada. On June 10th, 2025, a study information sheet and survey link were emailed through the GCEA listserv and reminder emails were sent approximately 2 weeks apart. Individual emails were collected through the GCEA Member Director Search (*Member Directory Search—Genetic Counselor Educators Association* [*GCEA*] 2025). A flyer with study information and the survey link was distributed at the annual GCEA conference July 14th–16th, 2025. The survey closed on August 8th, 2025.

### 2.2 Instrumentation

This cross‐sectional study utilized a quantitative survey (Appendix S1). Data were collected online using the secure, web‐based software Research Electronic Data Capture (REDCap) (Harris et al. 2019, 2009), provided through Indiana University. The survey was piloted for clarity and completeness by program leadership, GCs, and genetic counseling students at Indiana University's genetic counseling graduate program. Information about the study was provided on the first page of the survey, and participants provided informed consent by clicking “Continue.” Upon completion, participants had the opportunity to provide their email address in a separate survey to enter a random drawing to win one of five $50 Amazon gift cards. Responses were not linked to participants' email addresses. After the survey closed, winners for the gift card drawing were chosen using a random number generator, and gift cards were sent electronically via the email that was provided.

Participants were asked to choose their affiliated program from a drop‐down list. Once a program was selected, it was removed from the drop‐down list to ensure no more than one entry per program. All program names were removed from the dataset prior to analysis. Participant position title (select all that apply) and number of years affiliated with the program were collected. Descriptive information regarding the program was collected, including number of cohorts graduated, size of the incoming cohort, and the predominant method used to deliver didactic coursework (in‐person, virtual, or hybrid) across the curriculum rather than within any individual course. Information was also collected regarding if and what type of CGT treatments are offered (FDA‐approved and/or clinical trials) at or near the affiliated program. The option “I don't know” was provided for the latter questions.

The second section assessed CGT instruction included in the program. Participants were asked if their programs taught about CGT and if they considered CGT education important/will include it in the future with four response options provided. For programs that reported providing CGT education, a follow‐up question assessed how long the program had been regularly providing this education. To compare the importance of CGT education to other factors, programs that responded “important and currently included” were compared to all other responses combined.

**FIGURE 1:**
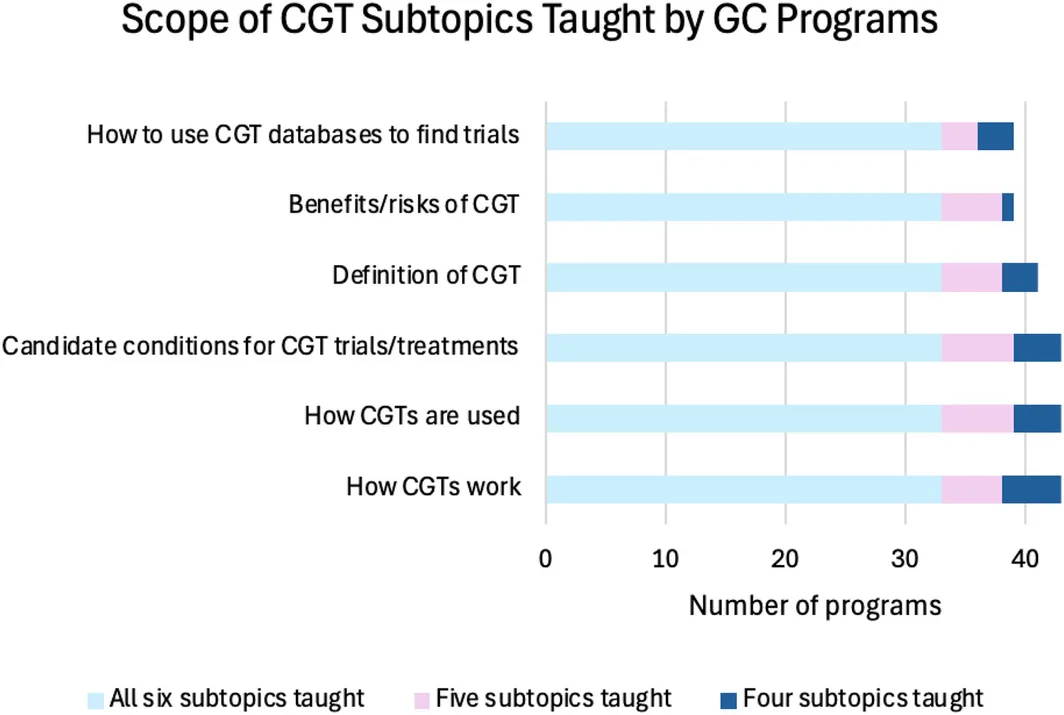
Scope of CGT subtopics covered in curriculum across programs.

To explore *what is taught*, questions assessed whether six CGT subtopics (see Figure 1) were taught by programs. Additional questions to assess *how CGT is taught* included: number of lectures a single cohort receives on CGT topics, number of readings assigned, attending departmental seminars, and attending grand rounds. We also asked participants to select any/all clinical rotations in which students participate that include instruction on CGT (metabolism, ocular, neurology, other, none).

**FIGURE 2:**
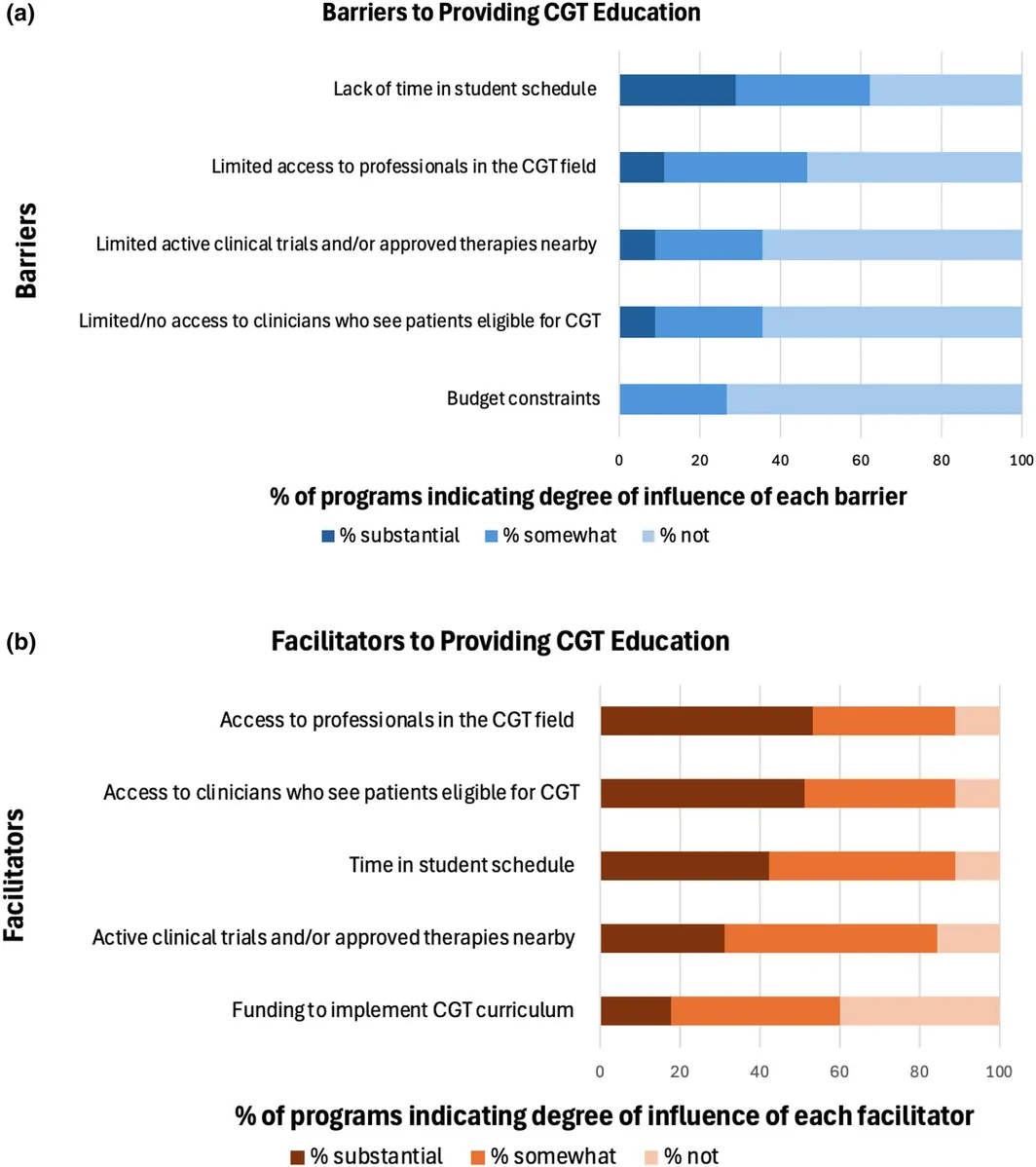
(a) Barriers for genetic counseling programs to implement education on CGT. (b) Facilitators for genetic counseling programs to implement education on CGT.

All participants were provided a list of five potential barriers in addition to “other” and asked to identify the degree to which each item was a barrier to implementing CGT education in the curriculum using a three‐point Likert scale (0 = “not a barrier,” 1 = “somewhat a barrier,” 2 = “substantial barrier”). The full list of barriers is provided in Figure 2. Barrier scores were created by summing the Likert responses, yielding scores ranging from 0 to 10. A list of five positive factors that may assist a program in implementing CGT education was similarly presented (0 = “not helpful,” 1 = “somewhat helpful,” 2 = “substantially helpful”). The full list of facilitators is provided in Figure 2. Similar to barriers, a facilitator score was calculated by summing the Likert responses for a score ranging from 0 to 10. The last question allowed for a free response to include comments on anything not covered in a previous section.

### 2.3 Data Analysis

Our primary goal was to describe specific training on CGT provided by genetic counseling training programs. Descriptive statistics are provided, including frequencies, mean, standard deviation (SD), and standard error (SE). To reduce the scope of testing, we limited analyses to key categorical and quantitative factors. Responses of “no” and “I don't know” were combined and compared to “yes” for each appropriate question. Quantitative variables included barrier and facilitator scores (described above).

A secondary goal was to identify barriers and facilitators associated with implementing this education. We used a Fisher's exact test to explore if there was an association between each individual barrier (“not” a barrier vs. “somewhat” or “substantial”) and: (1) whether or not programs had CGTs offered at or near their institution (yes vs. no) and (2) method of didactic training (in person vs. virtual/hybrid). Similar tests were employed for each facilitator. Due to the non‐normal distribution of the barrier and facilitator scores, a non‐parametric Wilcoxon test was utilized to explore if these scores were different (1) between programs that offered CGTs at or near their institution versus those that did not and (2) between the method of didactic training. Spearman's rank correlation coefficient was calculated to describe the correlation between the number of cohorts graduated, barrier score, and facilitator score. SAS v 9.4 was utilized for all analyses.

## 3 Results

### 3.1 Demographics

#### 3.1.1 Respondent Demographics

Leaders from 45 of the 65 contacted genetic counseling graduate programs consented and completed the survey (69.2%). The most frequently selected position was Program Director (71.1%). Most respondents (77.8%) have been affiliated with their respective program for 5 or more years. The first author manually confirmed at least one response was received from each NSGC region. All details are provided in Table S1.

#### 3.1.2 Program Demographics

The mean number of cohorts graduating since establishment was 21.9 (range 2–54 cohorts, SD = 16.4). Cohort size ranged from 5 to 27 students, with a mean of 10.9 students (Table 1). Most programs offered didactic courses in person (80%), with 15.6% offering hybrid, and the remaining were virtual. Most programs (82.2%) indicated that CGTs are offered at or near their institution. Of the 37 programs that have CGT treatments at or near their institution, 33 (89.2%) have both FDA‐approved therapies and clinical trials available locally (Table 1).

**TABLE 1:** Program demographics.

	Mean ± SD (range)
Number of cohorts graduated (*N* = 45)	21.91 ± 16.43 (2–54)
Size of incoming/current cohort (*N* = 45)	10.91 ± 4.72 (5–27)

	*N*	%
Didactic courses		
In person	36	80.0
Hybrid	7	15.56
Virtual	2	4.44
Cell or gene therapy treatments offered at or near institutions		
Yes	37	82.22
No	8	17.78
Type of cell or gene therapy treatments offered at or near institutions		
Both FDA approved therapies and clinical trials	33	89.19
I don't know	3	8.11
FDA approved therapies	1	2.70

*Note:* Type of cell or gene therapy treatments offered at or near institutions was only asked to respondents who have cell or gene therapy treatments offered at or near their institution (*N* = 37).

Abbreviations: FDA, U.S. Food and Drug Administration; *N*, number; SD, standard deviation.

### 3.2 Training

#### 3.2.1 Training Provided

Various questions explored the training that genetic counseling graduate programs provide on CGT. Most programs (43, 95.6%) reported that they currently offer training on CGT topics. One program reported they do not currently offer training on CGT topics, and one program reported that they were unsure if their program offers this type of training. Of those 43 programs, 55.8% have been providing this education for 5 years or less, and 44.2% have been providing this education for more than 5 years. Most programs (73.3%) had no plans to add more CGT training, while 26.7% of programs reported plans to either implement CGT training if not already in place or expand existing training.

#### 3.2.2 Modality of Training

While most programs offered CGT training, not all programs employed the same training modalities (Figure S1). One respondent that initially reported being unsure if their program taught CGT later selected several subtopics and training modalities used, indicating some CGT training is provided. This program's responses were included in analyses describing CGT subtopics and training modalities; therefore, these analyses were based on 44 programs. There was frequent overlap of CGT training methods used among the 44 reporting programs, with 29.6% (13 of 44 programs) offering all 4 training methods (CGT‐involved readings, lectures, grand rounds, and clinical rotations). Out of 44 programs, 41 programs offered readings as a mode of training, 31 programs provided clinical rotations that discuss CGT topics, 24 programs offered CGT‐involved grand rounds, and 23 programs offered lectures on CGT topics (Table 2). Common clinical rotation specialties involving CGT available to students included metabolism (48.9%), neurology (48.9%), and ocular rotations (31.1%). Fifteen programs (33.3%) selected “other” specialties with write‐in responses including clinical rotations in general genetics, hematology, sickle cell disease, lysosomal storage disease, hearing loss, pediatrics, and cardiology.

**TABLE 2:** Training methods.

			*N*	%
Readings	Yes	1–5 readings	31	70.45
		> 5 readings	10	22.73
	No		3	6.82
Clinical rotations	Yes	Metabolism rotation	22	48.89
		Neurology rotation	22	48.89
		Ocular rotation	14	31.11
		Other rotation	15	33.33
	No		13	29.55
Grand rounds	Yes	1–5 grand rounds	21	47.73
		> 5 grand rounds	3	6.82
	No		20	45.45
Departmental lectures	Yes	1–5 lectures	19	43.18
		> 5 lectures	4	9.09
	No		21	47.73

*Note:* CGT training methods used by genetic counseling graduate programs. Respondents were allowed to select more than one clinical rotation, so the total is greater than 100%.

#### 3.2.3 CGT Subtopics Taught

The majority of programs (33/44 programs, 75.0%) provide training on all 6 CGT subtopics, with 13.6% offering training on 5 subtopics and 11.4% offering training on 4 subtopics (Figure 1). All programs that did not have CGTs nearby still taught how CGTs work, how CGTs are used, candidate conditions for CGT trials and treatments, and the definition of CGT (Table 3). The least frequently covered subtopics were how to use clinical trial databases to find CGTs and the benefits and risks of CGTs (Figure 1).

**TABLE 3:** Cell and gene therapy subtopics.

Subtopic taught	Programs with nearby CGTs; *N* (%)	Programs without nearby CGTs; *N* (%)	All programs; *N* (%)
How CGTs work	35 (94.6%)	8 (100%)	43 (95.6)
How CGTs are used	35 (94.6%)	8 (100%)	43 (95.6)
Candidate conditions for CGT trials/treatments	35 (94.6%)	8 (100%)	43 (95.6)
Definition of CGT	33 (89.2%)	8 (100%)	41 (91.1)
Benefits/risks of CGTs	33 (89.2%)	6 (75%)	39 (86.7)
How to use CGT databases to find trials	33 (89.2%)	6 (75%)	39 (86.7)

*Note:* Cell and gene therapy (CGT) subtopics taught by programs with CGTs offered at or near their affiliated institution (*N* = 37) or with no CGTs offered at or near their institution (*N* = 8); combined no (*N* = 4) and I don't know (*N* = 4) responses.

### 3.3 Importance of CGT Education

Participants were asked how important CGT education is to their program. Out of 43 respondents, 36 (83.7%) indicated that they felt CGT education is important and is currently included in their program. Eight respondents (17.8%) selected that they feel CGT education is important and will be included in the future. One respondent (2.2%) indicated that they feel CGT education is important but lack resources to include it. No respondents indicated that they think CGT education is not important.

### 3.4 Influential Factors

#### 3.4.1 Barriers

Respondents reported the extent of five potential barriers on implementation of CGT training in their programs (Figure 2). The substantial barrier most frequently chosen was lack of time in students' schedules (28.9%) followed by limited access to professionals in the CGT field (11.1%). Free responses indicated additional barriers, including difficulty keeping up with the rapidly evolving science, lack of initiative to seek out experts who can teach on these topics, lack of familiarity with local resources, and difficulty knowing what content is appropriate and at what level to teach on CGT topics.

##### 3.4.1.1 Individual Barriers

**FIGURE 3:**
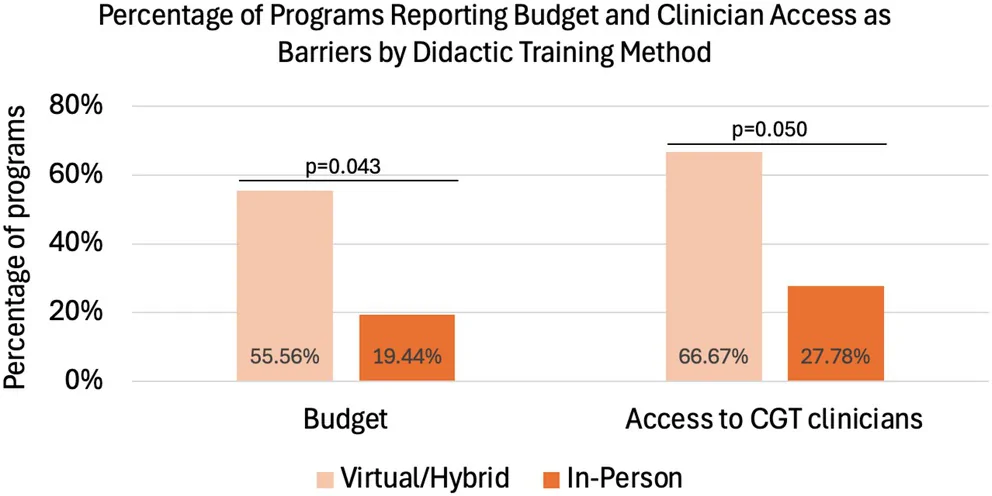
Percentage of programs reporting budget and clinician access as barriers by didactic training method.

Programs that teach their didactic coursework in a virtual or hybrid manner were more likely to select budget and access to clinicians in the CGT field as barriers to offering or implementing CGT education compared to programs that are in‐person (Fisher's exact *p* = 0.043, *p* = 0.050, respectively; Figure 3). There was no association of any individual barrier with whether or not programs had CGTs offered at or near their institution (Fisher's exact *p* > 0.24).

##### 3.4.1.2 Barrier Score

The mean barrier score was 2.8 (median = 2.0) with a standard deviation of 2.66 (see Figure S2). There was no correlation between the barrier score and the number of cohorts graduated (rho = 0.21, *p* = 0.18). The median number of barriers was not different between programs that currently include CGT versus programs that do not have plans to begin or add additional training on CGT or between programs where coursework is in‐person versus virtual or hybrid (both Wilcoxon *p* > 0.14).

#### 3.4.2 Facilitators

Respondents provided information on five positive factors that facilitate implementation of CGT education (Figure 2). Access to professionals in the CGT field was the greatest facilitator to implementing training (53.3% selected substantially helpful) followed by access to clinicians who see patients for CGT (51.1%). “Other” responses included having a webinar or recorded lecture(s) on the topics available to all genetic counseling programs, having curricular guidance, and having access to CGT centers of excellence.

##### 3.4.2.1 Individual Facilitators

There was no association between any individual facilitator and whether or not programs had CGTs offered at or near their institution, programs that currently include CGT versus programs that do not, or between programs where coursework is in‐person versus virtual or hybrid (all *p* > 0.22).

##### 3.4.2.2 Facilitator Score

The mean facilitator score was 6.1 (median = 7.0), with standard deviation = 2.29 (see Figure S2). There was no association between *facilitator score* and number of cohorts graduated (Wilcoxon *p* > 0.52). The median score was not different between programs that had CGTs offered at or near their institution compared to those that do not (Wilcoxon *p* > 0.28).

## 4 Discussion

To our knowledge, this is the first study investigating CGT training provided by genetic counseling graduate programs. We found that almost all programs currently offer education on CGT topics, with more than half having implemented such training within the past 5 years. Despite all participants reporting that CGT education is important, lack of time in student schedules and access to professionals in the CGT field were the most frequent barriers to programs including more training on CGT topics.

Although our study found that almost all training programs include some education on these topics, previous publications reported that GCs either do not remember education provided on CGT topics (Walsh et al. 2024) or do not feel their graduate program provided adequate training on CGT (Geiselman et al. 2024). Evidence suggests that initial instruction in the health professions does not always translate into sustained knowledge retention, and multiple studies document declines in knowledge after only a few months (Csaba et al. 2025; Custers and Ten Cate 2011; Greb et al. 2009). In contrast, simulation‐based learning (Alharbi et al. 2024), structured retrieval or distributed practice (Serra et al. 2025; Trumble et al. 2024), and other strategies (Albert et al. 2024) are associated with improved long‐term retention. These findings underscore the importance of adopting techniques to promote durable knowledge and skill retention around CGT treatment options.

The volume of available CGTs (*Q3 2025 Cell and Gene Therapy Sector Snapshot* 2025) and the invaluable treatments they provide (*Gene Therapy Program | FDA‐Approved Gene Therapies | Boston Children's Hospital* 2026) emphasize the importance of integrating CGT education into GCs' training. In fact, an international CGT working group recently outlined the need for standardized training in CGT (Maryamchik et al. 2025). While most respondents' programs include some CGT education in their curricula, CGT education is not currently a required educational content area by the ACGC, which leaves its inclusion and depth of coverage to the discretion of each program. Additionally, it is not clear whether CGT knowledge is explicitly tested among the “treatment and management” content of the American Board of Genetic Counseling certification exam. Because the accreditation standards specify a minimum board pass rate for programs' trainees, programs may be indirectly influenced to lend more resources to content covered on the board exam. The ABGC exam content outline is determined based on a practice analysis conducted every 5 years, with the last analysis performed in 2022. Given the recent growth of CGT options, the next practice analysis may reflect GCs' involvement in CGT discussions, which may in turn influence the ABGC exam contents and improve CGT education offered by GC training programs.

Almost all programs indicated that lack of time in the students' schedule was a barrier to adding CGT curriculum. This is not surprising, as a recent qualitative study reported that this was the biggest challenge in making changes to the genetic counseling training curriculum (Vercruyssen et al. 2025). Several options could be implemented into training that would not increase the time burden; for example, incorporating CGT mechanisms into molecular genetics lectures, adding gene therapy counseling scenarios into simulation exercises, including a CGT vignette in risk assessment or ethics modules, or, as suggested by Trumble et al. (2024), repeated exposures to CGT in the form of brief “CGT clinical updates” embedded into classes.

The most frequently cited facilitator to implementing CGT training was access to professionals in the CGT field. Weaving collaboration with professionals outside the training program into the educational process has been shown to improve several outcome measures related to patient health (Cadet et al. 2024). A recent qualitative study described non‐academic clinicians helping to develop and teach curriculum in a health‐related training program. Despite the majority agreeing that this would add to their workload, more than three‐quarters indicated they would be willing to engage in student training (Kenny et al. 2023). These studies suggest that CGT professionals may be willing to partner with nearby training programs, leading to improved educational outcomes. At present, no centralized, publicly searchable directory of CGT professionals in North America exists to facilitate such engagement. To address this gap, we compiled a curated, geographically organized directory of major CGT centers near academic centers in the United States and Canada (Table S2) to facilitate collaboration between CGT professionals and genetic counseling training programs.

### 4.1 Strengths and Limitations

One of the strengths of this study is the inclusion of information from more than two‐thirds of genetic counseling graduate programs in the United States and Canada, including responses from programs in each region. Options for narrative responses provided novel insights into factors that helped or hindered implementation of CGT training into the curriculum. While this study included responses from the majority of genetic counseling programs, it did not encompass all program perspectives. Therefore, the results may not be generalizable to all genetic counseling programs. Participants also self‐reported on behalf of their program, which could have introduced self‐reporting bias. Additionally, we did not assess whether all students are required to complete every rotation specialty that includes CGT content, and therefore some programs may offer CGT‐related training in elective rotations that not all students complete. We acknowledge that some survey questions may have been interpreted differently than intended. Lastly, this was a cross‐sectional study; therefore, these findings do not represent trends across time, which is especially important given the continuously changing field of CGT.

### 4.2 Future Directions

This study provides a platform for further discussion regarding the utility of a NSGC practice guideline about CGTs for GCs; however, future research is needed to explore the potential impact of improving CGT training in education. A qualitative study could help us understand challenges programs are facing individually and if shared educational materials would be beneficial, while a needs‐assessment survey could explore the benefits of different types of education, both at the graduate and professional levels. Additionally, programs' use of teaching strategies that contribute to long‐term retention could be helpful to assess in future CGT education research.

## 5 Conclusion

This is the first study to explore CGT education offered by genetic counseling graduate programs and to assess factors that help and hinder the implementation of this education. While most GC programs incorporate some training on CGT topics, variability in training modalities and topics may contribute to GCs feeling underprepared to discuss these topics with patients. With the increase in CGT clinical trials and FDA‐approved CGT treatments, GCs should be knowledgeable about these therapies to best serve their patients. Future actions are needed to strengthen this education, and it will take a collaborative effort between GC educators and CGT experts to better integrate CGT knowledge into GC training curriculum.

## Author Contributions

**Mariel Sebby:** conceptualization, writing – original draft, writing – review and editing, methodology, funding acquisition. **Lily McConachie:** conceptualization, writing – review and editing, methodology, supervision. **Leah Wetherill:** conceptualization, writing – review and editing, formal analysis, methodology, supervision. **Paula Delk:** writing – review and editing, supervision. **Emily L. Hopewell:** conceptualization, writing – review and editing, methodology, supervision.

## Funding

This work was supported by internal funds from the Indiana University School of Medicine Department of Medical and Molecular Genetics.

## Ethics Statement

*Human Studies and Informed Consent*: This study was deemed exempt by the Indiana University Institutional Review Board (Protocol #27284). Consent was implied upon entering the survey.

*Animal Studies*: The authors have nothing to report.

## Conflicts of Interest

The authors declare no conflicts of interest.

## Supporting information


**Appendix S1:** Survey used in study data collection.

**Figure S1:** Venn diagram of training modalities and their overlaps showing the number of genetic counseling programs utilizing each modality to teach on CGT topics. The most common selection was having all four of the training modalities (13/44, 28.9%). The next most common selection was providing training with readings and clinical rotations.

**Figure S2:** Distribution of barrier and facilitator scores.

**Table S1:** Respondent demographics.

**Table S2:** CGT training engagement director—Institutional level resource.

## Data Availability

De‐identified data supporting the findings of this study are available from the corresponding author upon reasonable request.
