# Supplementary material for: Teaching Cell and Gene Therapy in Genetic Counseling Programs: Survey Findings and Suggested Practical Resources for Integration
Source: J Genet Couns. 2026 Sep 22;35(5):e70295. doi: 10.1002/jgc4.70295 (PMC13596064; doi:10.1002/jgc4.70295)
Supplement: Supplementary file 1 [file JGC4-35-0-s001.docx]

**Supplemental Figure 1.** Venn diagram of training modalities and their overlaps showing the number of genetic counseling programs utilizing each modality to teach on CGT topics. The most common selection was having all four of the training modalities (13/44, 28.9%). The next most common selection was providing training with readings and clinical rotations.
