# Supplementary material for: Teaching Cell and Gene Therapy in Genetic Counseling Programs: Survey Findings and Suggested Practical Resources for Integration
Source: J Genet Couns. 2026 Sep 22;35(5):e70295. doi: 10.1002/jgc4.70295 (PMC13596064; doi:10.1002/jgc4.70295)
Supplement: Supplementary file 2 [file JGC4-35-0-s002.docx]

**Supplemental Appendix 1:** Survey used in study data collection.

Cell and Gene Therapy in Genetic Counseling Education

**Demographics:**

This section of the survey is to collect information on the genetic counseling graduate program with which you are affiliated. Please answer the questions on behalf of your program, not your individual experience. Program selection is solely used to ensure only one response per program. All data will be deidentified prior to analysis.

1. Has your accredited genetic counseling training program graduated at least one cohort?
   a. Yes
   b. No

Message that will appear if not eligible (selecting “No”): Unfortunately, your training program does not meet the inclusion criterion of having graduated at least one cohort. Thank you for your time!

2. What accredited genetic counseling graduate program are you affiliated with?

If you represent a program that is not found in the list, the survey has been completed on behalf of your program. Thank you kindly for taking the time to participate in this survey.

1. Arizona State University
2. Augustana University
3. Bay Path University
4. Baylor College of Medicine
5. Boise State University
6. Boston University Chobanian & Avedisian School of Medicine
7. Case Western Reserve University
8. Charles R. Drew University of Medicine and Science
9. Columbia University
10. Emory University School of Medicine
11. Geisinger College of Health Sciences
12. Indiana University
13. Johns Hopkins University/National Human Genome Research Institute
14. Kean University
15. Keck Graduate Institute
16. Loma Linda University – School of Medicine
17. Long Island University (LIU) Post
18. McGill University
19. Medical College of Wisconsin
20. Medical University of South Carolina
21. MGH Institute of Health Professions
22. Mt. Sinai School of Medicine
23. Northwestern University Medical School
24. Rutgers University
25. Sarah Lawrence College
26. Southern California University
27. Stanford University
28. The Ohio State University
29. Thomas Jefferson University
30. University at Buffalo
31. University of Alabama at Birmingham
32. University of Arizona
33. University of Arkansas Medical Sciences
34. University of British Columbia
35. University of California, Irvine
36. University of California, Los Angeles
37. University of California, San Francisco
38. University of Cincinnati College of Medicine
39. University of Colorado Denver
40. University of Connecticut
41. University of Kansas School of Health Professionals
42. University of Manitoba
43. University of Maryland School of Medicine
44. University of Michigan
45. University of Minnesota
46. University of Nebraska Medical Center
47. University of North Carolina at Greensboro
48. University of Pennsylvania
49. University of Pittsburgh
50. University of Rochester
51. University of South Carolina
52. University of South Florida
53. University of Texas Graduate School of Biomedical Sciences at Houston
54. University of Texas Southwestern Medical Center
55. University of Toronto
56. University of Utah
57. University of Washington
58. University of Wisconsin-Madison
59. Vanderbilt University
60. Virginia Commonwealth University
61. Wake Forest University
62. Washington University in St. Louis
63. Wayne State University
64. Weill Cornell Medical College
65. Xavier University of Louisiana
66. Other (fill in the blank)

If “Other (fill in the blank)” is selected: What accredited genetic counseling graduate program are you affiliated with? Other (fill in the blank):

3. How many classes/cohorts has your genetic counseling training program graduated since its establishment?

Respondents can select from a drop-down list containing options of 0 - 60 or “Other”.

If “Other” is selected: How many classes/cohorts has your genetic counseling training program graduated since its establishment? Other:

4. What is the size of your current / incoming cohort?
   a. Fill in the blank
5. In what year was your program first accredited by the Accreditation Council for Genetic Counseling (ACGC) or American Board of Genetic Counseling (ABGC)?
   a. Fill in the blank
6. How many total years have you been working for the program with which you are currently affiliated?
   a. Less than 1 year
   b. 1 – 2.9 years
   c. 3 – 4.9 years
   d. 5 or more years
7. What is the title of your position at your genetic counseling training program? Select all that apply.
   a. Program Director
   b. Assistant / Associated Program Director
   c. Assistant / Associated / Full Professor
   d. Program Coordinator
   e. Medical Director
   f. Field Work Coordinator
   g. Research Coordinator
   h. Other / Fill in the blank

If “Other / Fill in the blank)” is selected: Other / Fill in the blank: (free text box)

8. How does your genetic counseling training program offer its didactic courses?
   a. In person
   b. Virtual
   c. Hybrid
9. Are cell or gene therapy treatments offered at or near your institution?
   a. Yes
      i. Please specify what cell or gene therapy treatments are offered at or near your institution.
         1. FDA approved therapies
         2. Clinical trials
         3. I don’t know
         4. Both approved and clinical trials
   b. No
   c. I don’t know

**Cell and Gene Therapy Education:**

This section aims to identify if cell and gene therapy topics are taught at your affiliated program.

**Cell and gene therapies** are emerging treatments that either replace or modify cells or genes to treat disease. Cell therapy uses living cells to treat a patient while gene therapy targets the genetic material within cells to treat a patient.

**Clinical trials** are currently evaluating cell and gene therapy treatments for several genetic conditions that, if approved by the US Food and Drug Administration (FDA), could become successful treatment options for patients with genetic conditions. Currently, the FDA has approved at least 43 cell and gene therapies within the United States. These approved therapies treat several genetic conditions including sickle cell disease, Duchenne muscular dystrophy, and many more.

10. Does your program include training on cell and gene therapy?
    a. Yes
    b. No
    c. I don’t know
11. How many years has your affiliated program regularly been providing this education?
    a. Less than 1 year
    b. 1-3 years
    c. 4-5 years
    d. More than 5 years
12. How important is CGT education to your program?
    a. Not important and no plans to include CGT in the future
    b. Important but lack resources to include CGT in the future
    c. Important and will include CGT in the future
    d. Important and currently include CGT
13. Is there any other information you wish to include on the importance of CGT education to your program?
    a. Free text box

**Cell and Gene Therapy Education – Covered Content:**

This section aims to identify what type of didactic education on cell and gene therapy topics is provided at your affiliated program.

**Cell and gene therapies** are emerging treatments that either replace or modify cells or genes to treat disease. Cell therapy uses living cells to treat a patient while gene therapy targets the genetic material within cells to treat a patient.

**Clinical trials** are currently evaluating cell and gene therapy treatments for several genetic conditions that, if approved by the US Food and Drug Administration (FDA), could become successful treatment options for patients with genetic conditions. Currently, the FDA has at least 43 approved cell and gene therapies within the United States. These approved therapies treat several genetic conditions including sickle cell disease, Duchenne muscular dystrophy, and many more.

14. Please answer the following questions about the training provided to your genetic counseling students.

(Matrix, 3-point Likert scale)

| Does your affiliated program… | **Yes** | **No** | **I don’t know** |
| --- | --- | --- | --- |
| Teach the definition of cell and gene therapies |  |  |  |
| Teach how cell and gene therapies work |  |  |  |
| Teach how cell and gene therapies are used |  |  |  |
| Teach about the benefits and risks associated with cell and gene therapies |  |  |  |
| Teach about the types of genetic conditions that might qualify for cell and gene therapy trials / treatments |  |  |  |
| Teach how to use clinical trial databases to find suitable trials for patients |  |  |  |

15. Do you have plans to begin or add additional training on cell and gene therapy?

* Each option once chosen will pop up the same free response question with the text: Please provide any additional details you wish to add on this question.

a. Yes
b. No
c. I don’t know

**Cell and Gene Therapy Education – Instructional Methods:**

This section aims to identify how cell and gene therapy topics are taught at your affiliated program.

**Cell and gene therapies** are emerging treatments that either replace or modify cells or genes to treat disease. Cell therapy uses living cells to treat a patient while gene therapy targets the genetic material within cells to treat a patient.

**Clinical trials** are currently evaluating cell and gene therapy treatments for several genetic conditions that, if approved by the US Food and Drug Administration (FDA), could become successful treatment options for patients with genetic conditions. Currently, the FDA has at least 43 approved cell and gene therapies within the United States. These approved therapies treat several genetic conditions including sickle cell disease, Duchenne muscular dystrophy, and many more.

**Clinical rotations** are structured training experiences, either observational or participation-based, designed to provide knowledge and exposure to various areas of genetic counseling.

**Lectures** consist of traditional, structured instructional sessions provided to each cohort typically in a classroom format. This may include professional experts in the field invited to speak on cell and gene therapy topics.

**Departmental seminars** consist of educational sessions outside the typical classroom setting.

16. How is cell and gene therapy taught within your program?
    a. Lectures: How many lectures will a single cohort receive on cell and gene therapy topics?
       i. 0
       ii. 1-5
       iii. More than 5
    b. Readings: How many readings will a single cohort be assigned on cell and gene therapy topics?
       i. 0
       ii. 1-5
       iii. More than 5
    c. Clinical rotations: Do students participate in clinical rotations in which they learn about cell and gene therapy?
       i. Yes
          1. If yes, what rotations discuss cell and gene therapy? Select all that apply.
             a. Metabolism
             b. Ocular
             c. Neurology
             d. Other

i. Please describe the other rotation(s) that discuss cell and gene therapy. (Free text box)

ii. No
d. Departmental seminars: Do students attend departmental seminars in which they learn about cell and gene therapy?
   i. Yes
      1. If yes, how many lectures are attended?
         a. 1-5
         b. More than 5
   ii. No
e. Grand rounds: Do students attend grand rounds in which they learn about cell and gene therapy?
   i. Yes
      1. If yes, how many grand rounds are attended?
         a. 1-5
         b. More than 5
   ii. No
f. Please describe other opportunities for students to learn about cell and gene therapy if applicable.
   i. Free text box

**Cell and Gene Therapy Education – Barriers:**

This section aims to identify barriers that may prohibit a program from implementing cell and gene therapy education in their curriculum.

**Cell and gene therapies** are emerging treatments that either replace or modify cells or genes to treat disease. Cell therapy uses living cells to treat a patient while gene therapy targets the genetic material within cells to treat a patient.

**Clinical trials** are currently evaluating cell and gene therapy treatments for several genetic conditions that, if approved by the US Food and Drug Administration (FDA), could become successful treatment options for patients with genetic conditions. Currently, the FDA has at least 43 approved cell and gene therapies within the United States. These approved therapies treat several genetic conditions including sickle cell disease, Duchenne muscular dystrophy, and many more.

17. To what degree are the following factors a barrier in implementing cell and gene therapy education?

|  | Not a barrier | Somewhat a barrier | Substantial barrier |
| --- | --- | --- | --- |
| Budget constraints |  |  |  |
| Lack of time in student schedule |  |  |  |
| Limited active clinical trials and/or approved therapies nearby |  |  |  |
| Limited access to professionals in the cell and gene therapy field |  |  |  |
| Limited/no access to clinicians who see patients eligible for cell and gene therapy |  |  |  |

18. Are there any other barriers not included in this list that your program faces in implementing cell and gene therapy education?
    a. Yes
       i. Please describe other barriers indicating if they are not a barrier, somewhat a barrier, or a substantial barrier. (Free text box)
    b. No

**Cell and Gene Therapy Education – Facilitators:**

This section aims to identify positive factors that may assist a program in implementing cell and gene therapy education in their curriculum.

**Cell and gene therapies** are emerging treatments that either replace or modify cells or genes to treat disease. Cell therapy uses living cells to treat a patient while gene therapy targets the genetic material within cells to treat a patient.

**Clinical trials** are currently evaluating cell and gene therapy treatments for several genetic conditions that, if approved by the US Food and Drug Administration (FDA), could become successful treatment options for patients with genetic conditions. Currently, the FDA has at least 43 approved cell and gene therapies within the United States. These approved therapies treat several genetic conditions including sickle cell disease, Duchenne muscular dystrophy, and many more.

19. To what degree are the following factors helpful (or would be helpful) in implementing cell and gene therapy education?

|  | Not helpful | Somewhat helpful | Substantially helpful |
| --- | --- | --- | --- |
| Funding to implement CGT curriculum |  |  |  |
| Time in student schedule |  |  |  |
| Active clinical trials and/or approved therapies nearby |  |  |  |
| Access to professionals in the cell and gene therapy field |  |  |  |
| Access to clinicians who see patients eligible for cell and gene therapy |  |  |  |

20. Are there any other positive factors not included in this list that your program finds helpful in implementing cell and gene therapy education?
    a. Yes
       i. Please describe other positive factors indicating if they are not helpful, somewhat helpful, or substantially helpful. (Free text box)
    b. No
21. Is there anything else you wish to add that was not covered in this survey? Please let us know.
    a. Free text box
