# Supplementary material for: Teaching Cell and Gene Therapy in Genetic Counseling Programs: Survey Findings and Suggested Practical Resources for Integration
Source: J Genet Couns. 2026 Sep 22;35(5):e70295. doi: 10.1002/jgc4.70295 (PMC13596064; doi:10.1002/jgc4.70295)
Supplement: Supplementary file 3 [file JGC4-35-0-s003.docx]

| Supplemental Table 1: Respondent Demographics | | |
| --- | --- | --- |
|  | N | % |
| Position Titles |  |  |
| Program Director | 32 | 71.11 |
| Assistant/Associate Program Director | 8 | 17.78 |
| Assistant/Associate/Full Professor | 3 | 6.67 |
| Field Work Coordinators | 2 | 4.44 |
| Research Coordinator | 1 | 2.22 |
| Other | 3 | 6.67 |
| Number of Position Titles Selected |  |  |
| 1 position | 41 | 91.1 |
| 2 positions | 3 | 6.67 |
| 3 positions | 1 | 2.22 |
| Total Years Affiliated with Program |  |  |
| 5 or more years | 35 | 77.78 |
| 3-4.9 years | 6 | 13.33 |
| 1-2.9 years | 3 | 6.67 |
| Less than 1 year | 1 | 2.22 |

**Supplemental Table 1:** Respondent Demographics. Respondents were able to select more than one position title; therefore, the total for Position Titles exceeds 100%.
