# Supplementary material for: Teaching Cell and Gene Therapy in Genetic Counseling Programs: Survey Findings and Suggested Practical Resources for Integration
Source: J Genet Couns. 2026 Sep 22;35(5):e70295. doi: 10.1002/jgc4.70295 (PMC13596064; doi:10.1002/jgc4.70295)
Supplement: Supplementary file 4 [file JGC4-35-0-s004.docx]

Distribution of Barrier and Facilitator Scores

Number of Programs

| Score | Barrier score | Facilitator score |
| --- | --- | --- |
| 0 | 13 | 1 |
| 1 | 5 | 2 |
| 2 | 7 | 0 |
| 3 | 6 | 2 |
| 4 | 4 | 4 |
| 5 | 2 | 9 |
| 6 | 4 | 4 |
| 7 | 2 | 12 |
| 8 | 0 | 6 |
| 9 | 2 | 2 |
| 10 | 0 | 3 |

Supplemental Figure 2. Distribution of barrier and facilitator scores.
