# Supplementary material for: Teaching Cell and Gene Therapy in Genetic Counseling Programs: Survey Findings and Suggested Practical Resources for Integration
Source: J Genet Couns. 2026 Sep 22;35(5):e70295. doi: 10.1002/jgc4.70295 (PMC13596064; doi:10.1002/jgc4.70295)
Supplement: Supplementary file 5 [file JGC4-35-0-s005.docx]

| Supplemental Table 2: CGT Training Engagement Director – Institutional Level Resource | | | | | |
| --- | --- | --- | --- | --- | --- |
| State/ Province | **City** | **Institution** | **Program Name** | **Primary CGT Modality** | **Program Website** |
| BC | Vancouver | BC Cancer | Cancer immunotherapy Program | CAR-T / Cell Therapy | <https://www.bccrc.ca/dept/drc/programs/bc-cancer-immunotherapy-program> |
| CA | Davis | UC Davis | Gene Therapy Center | Gene Therapy | <https://genetherapy.ucdavis.edu/> |
| CA | Duarte | City of Hope | Cell & Gene Therapy Programs | CAR-T / Broad CGT | <https://www.cityofhope.org> |
| CA | Los Angeles | UCLA | Jonsson Comprehensive Cancer Center – Cell Therapy | CAR-T / Cell Therapy | <https://www.uclahealth.org> |
| CA | San Francisco | UCSF | Alpha Clinic (Cell & Gene Therapy) | Cell & Gene Therapy Trials | <https://alphaclinic.ucsf.edu/> |
| CA | Stanford | Stanford University | Division of Human Gene Therapy | Gene Therapy | <https://med.stanford.edu/genetherapy.html> |
| CO | Aurora | University of Colorado Anschutz | Gates Biomanufacturing Facility / CGT | Cell & Gene Therapy | <https://medschool.cuanschutz.edu> |
| DC | Washington | Children’s National Hospital | Gene Therapy Program | Gene Therapy | <https://childrensnational.org> |
| FL | Gainesville | University of Florida | Powell Gene Therapy Center | Gene Therapy | <https://genetherapy.ufl.edu/> |
| FL | Miami | University of Miami | Transplantation and Cellular Therapy | Cell Therapy | https://med.miami.edu/departments/medicine/divisions/cellular-therapy |
| FL | Orlando | Nemours Children’s Hospital | Gene Therapy Program | Gene Therapy | <https://www.nemours.org> |
| FL | Tampa | Moffitt Cancer Center | Cellular Immunotherapy Program | CAR-T / Immunotherapy | <https://moffitt.org> |
| GA | Atlanta | Emory University | Winship Cancer Institute – Cell Therapy | CAR-T / Cell Therapy | <https://winshipcancer.emory.edu> |
| IA | Iowa City | University of Iowa | Gene Therapy Center | Gene Therapy | <https://medicine.uiowa.edu> |
| IL | Chicago | Northwestern University | Mathews Center for Cellular Therapy | CAR-T / Cell Therapy | <https://www.feinberg.northwestern.edu> |
| IL | Chicago | University of Chicago | Cell Therapy Program | CAR-T / Cell Therapy | <https://www.uchicagomedicine.org> |
| IN | Indianapolis | Indiana University School of Medicine | Brown Center for Immunotherapy | Cell Therapy / Immunotherapy | <https://medicine.iu.edu> |
| KS | Kansas City | KU Medical Center | Midwest Stem Cell Therapy Center | Cell Therapy | https://www.kumc.edu/research/midwest-stem-cell-therapy-center.html |
| LA | New Orleans | Tulane University | Center for Stem Cell Research & Regenerative Medicine | Gene & Cell Therapy | <https://medicine.tulane.edu> |
| MA | Boston | Boston Children’s Hospital | Gene Therapy Program | Gene Therapy | <https://www.childrenshospital.org> |
| MA | Boston | Dana-Farber Cancer Institute | Cellular Immunotherapy Program | CAR-T / Cell Therapy | <https://www.dana-farber.org> |
| MA | Boston | Mass General Brigham | Grousbeck Gene Therapy Center | Gene Therapy | <https://www.massgeneralbrigham.org> |
| MA | Worcester | UMass Chan Medical School | Horae Gene Therapy Center | Gene Therapy | <https://www.umassmed.edu/gene-therapy/> |
| MD | Baltimore | Johns Hopkins University | Kimmel Cancer Center – Cell Therapy | CAR-T / Cell Therapy | <https://www.hopkinsmedicine.org> |
| MI | Ann Arbor | University of Michigan | Cellular Therapy Program | CAR-T / Cell Therapy | <https://www.rogelcancercenter.org> |
| MN | Minneapolis | University of Minnesota | Gene Therapy Center | Gene Therapy | <https://med.umn.edu> |
| MN | Rochester | Mayo Clinic | CAR-T Cell Therapy Program | CAR-T Cell Therapy | <https://www.mayoclinic.org> |
| NC | Chapel Hill | UNC Chapel Hill | Gene Therapy Center | Gene Therapy | <https://www.med.unc.edu/gene-therapy/> |
| NC | Durham | Duke University | Cellular Therapy Program | CAR-T / Cell Therapy | <https://www.dukecancerinstitute.org> |
| NJ | Glassboro | Rowan University | Cell & Gene Therapy Center | Gene Therapy | <https://sites.rowan.edu/cgt/> |
| NY | New York | Memorial Sloan Kettering Cancer Center | Cell Therapy and Immunotherapy | CAR-T / Cell Therapy | <https://www.mskcc.org> |
| OH | Cincinnati | Cincinnati Children’s | Applied Gene and Cell Therapy Center | Gene Therapy / Cell Therapy | https://www.cincinnatichildrens.org/research/divisions/a/applied-gene-cell-therapy |
| OH | Cleveland | Cleveland Clinic | Cellular Therapy Program | CAR-T / Cell Therapy | <https://my.clevelandclinic.org> |
| OH | Columbus | Nationwide Children’s Hospital | Center for Gene Therapy | Gene Therapy | <https://www.nationwidechildrens.org> |
| OH | Columbus | The Ohio State University Comprehensive Cancer Center | Cell Therapy Laboratory | CAR-T / Cell Therapy | https://cancer.osu.edu/for-patients-and-caregivers/learn-about-cancers-and-treatments/innovation-at-the-james/immunotherapy/cell-therapy-program/cell-therapy-lab |
| ON | Toronto | University Health Network / University of Toronto | Center for Cell and Vector Production | Cell & Gene Therapy | <https://www.uhnresearch.ca/service/centre-cell-and-vector-production-ccvp?utm> |
| PA | Philadelphia | Children’s Hospital of Philadelphia (CHOP) | Cell & Gene Therapy Collaborative | Gene Therapy | <https://www.chop.edu> |
| PA | Philadelphia | University of Pennsylvania | Center for Cellular Immunotherapies | CAR-T / Cell Therapy | <https://www.med.upenn.edu/cellcenter/> |
| QC | Montreal | McGill University Health Centre | Cell Therapy Laboratory | CAR-T / Cell Therapy | <https://muhc.ca/car-t> |
| SC | Charleston | Medical University of South Carolina | Center for Cellular Therapy | Cell Therapy | https://medicine.musc.edu/cellular-therapy |
| TX | Houston | Baylor College of Medicine | Center for Cell & Gene Therapy | Cell & Gene Therapy | <https://www.bcm.edu> |
| TX | Houston | MD Anderson Cancer Center | Cell Therapy Program | CAR-T / Cell Therapy | <https://www.mdanderson.org> |
| WA | Seattle | Fred Hutchinson Cancer Center | Immunotherapy / Cell Therapy Programs | CAR-T / Cell Therapy | <https://www.fredhutch.org> |
| WA | Seattle | Seattle Children’s Hospital | Gene Therapy Program | Gene Therapy | <https://www.seattlechildrens.org> |
| WI | Madison | University of Wisconsin – Madison | Program for Advanced Cell Therapy | CAR-T / Cell Therapy | https://pact.wisc.edu/ |
| WI | Milwaukee | Medical College of Wisconsin Cancer Center | Cell Therapy Laboratory Shared Resource | CAR-T / Cell Therapy | https://cancer.mcw.edu/researchers-and-clinicians/shared-resources/cell-therapy-ctsr |

**Supplemental Table 2: CGT Training Engagement Director – Institutional Level Resource**

This table lists major U.S. and Canadian academic Cell and Gene Therapy (CGT) centers. It is intended to facilitate collaboration between CGT professionals and Genetic Counseling training programs. All institution websites were publicly available at time of compilation. This list is not exhaustive but meant to be an initial resource.

Abbreviations: CGT: cell and gene therapy
